# P-313. Black Women’s Experiences on Long-Acting Cabotegravir for PrEP: Interim Patient Findings from the EBONI Study

**DOI:** 10.1093/ofid/ofaf695.532

**Published:** 2026-01-11

**Authors:** Katherine L Nelson, Zandraetta Tims-Cook, Helena Kwakwa, Megan Dieterich, Tammeka Evans, Alftan Dyson, Neetu Badhoniya, Heidi Swygard, Michael Aboud, Kenneth Sutton, Denise Sutherland-Phillips, Dhuly Chowdhury, Nicole Mack, Piotr Budnik, Kimberley Brown, Maggie Czarnogorski, Nanlesta Pilgrim

**Affiliations:** ViiV Healthcare, Philadelphia, PA; Faebris Medical & Community Education, Atlanta, Georgia; Philadelphia Department of Public Health, Philadelphia, Pennsylvania; Whitman-Walker Institute, Washington DC, District of Columbia; ViiV Healthcare, Philadelphia, PA; ViiV Healthcare, Philadelphia, PA; GSK, London, England, United Kingdom; ViiV Healthcare, Philadelphia, PA; ViiV Healthcare, Philadelphia, PA; ViiV Healthcare, Philadelphia, PA; ViiV Healthcare, Philadelphia, PA; RTI International, Research Triangle Park, North Carolina; RTI International, Research Triangle Park, North Carolina; ViiV Healthcare, Philadelphia, PA; ViiV Healthcare, Philadelphia, PA; ViiV Healthcare, Philadelphia, PA; ViiV Healthcare, Philadelphia, PA

## Abstract

**Background:**

Black women represent ∼50% of new HIV diagnoses among transgender and cisgender US women, yet HIV PrEP uptake remains low. EBONI, a Phase IV study, evaluates long-acting cabotegravir (CAB LA) for PrEP delivery to Black women in US EHE areas. We present interim (4-month) experiences of access, delivery, and satisfaction with CAB LA among Black cis- and transgender women receiving CAB LA.

**Figure:**
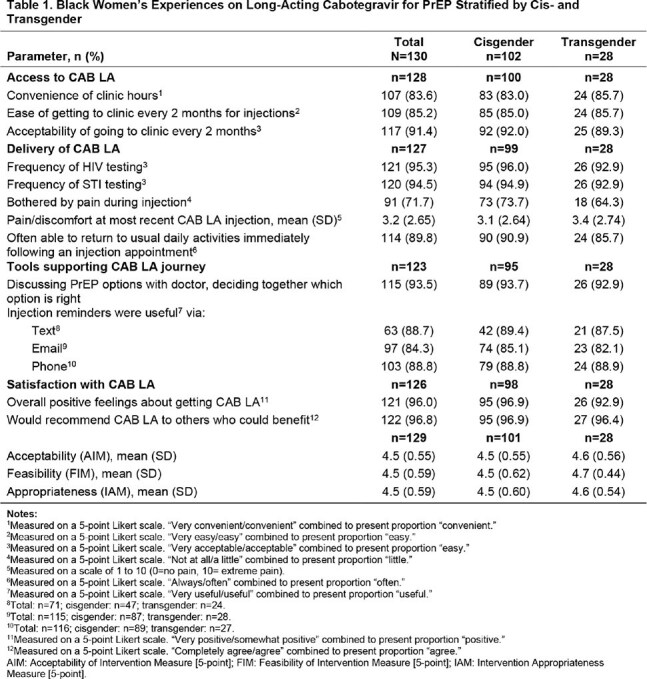

**Methods:**

From April 2023–February 2025, 130 Black women from 19 clinics completed interim surveys. Descriptive statistics were generated.

**Results:**

Black women were mostly cisgender (78%) and non-Hispanic (94%); mean age was 37.8 years (SD=10.5). Most women who had sex in the last 6 months (88%) had no sexual partners with HIV (62%) and did not have sexual partners taking PrEP (63%). CAB LA was accessible to Black women, with 84% reporting convenient clinic hours, 85% reporting no difficulty getting to the clinic every two months, and 91% finding it acceptable to come to the clinic every two months for injections (Table 1). Black women reported a positive delivery experience for CAB LA, with 95% of women reporting that the frequency of HIV and STI testing was acceptable, 72% reporting “not at all” or “a little” bothered by pain during injection, low ratings of injection pain (3.2 out of 10, SD=2.65), and 90% reporting they were “often”/“always” able to return to usual daily activities immediately following an injection. Some key supporting actions were useful to Black women during their CAB LA journey including discussions with their doctors about the pros and cons of different PrEP options and deciding together which one was right for them (94%) and injection appointment reminders via text (89%), email (84%), or phone (89%). Black women were satisfied with CAB LA; 96% reported overall positive feelings about CAB LA and 97% agreed they would recommend CAB LA to other people who could benefit. Overall, Black women found CAB LA to be acceptable, feasible, and appropriate (mean: > 4.5/5).

**Conclusion:**

Black women were highly satisfied with CAB LA after four months. They found CAB LA easy to access at their clinics, had positive experiences with CAB LA delivery, and highlighted the importance of shared decision-making with their providers on their CAB LA journey.

**Disclosures:**

Katherine L. Nelson, PhD, MPH, ViiV Healthcare: Employee|ViiV Healthcare: Stocks/Bonds (Private Company) Zandraetta Tims-Cook, MD, Syneos: Support for the present publication|ViiV Healthcare: Advisor/Consultant|ViiV Healthcare: Support for the present publication and paid speaker fees for products, including Apretude Megan Dieterich, MPH, MMSc, PA-C, ViiV Healthcare: Grant/Research Support Tammeka Evans, MoP, ViiV Healthcare: Employee|ViiV Healthcare: Stocks/Bonds (Private Company) Alftan Dyson, PharmD, ViiV Healthcare: Employee|ViiV Healthcare: Stocks/Bonds (Private Company) Neetu Badhoniya, PhD, GSK: Employee|GSK: Stocks/Bonds (Private Company) Heidi Swygard, MD, ViiV Healthcare: Employee|ViiV Healthcare: Stocks/Bonds (Private Company) Michael Aboud, MBChB, MRCP, ViiV Healthcare: Employee|ViiV Healthcare: Stocks/Bonds (Private Company) Kenneth Sutton, MA, ViiV Healthcare: Employee|ViiV Healthcare: Stocks/Bonds (Private Company) Denise Sutherland-Phillips, MD, ViiV Healthcare: Employee|ViiV Healthcare: Stocks/Bonds (Private Company) Piotr Budnik, MBBCh FCP(SA), ViiV Healthcare: Employee|ViiV Healthcare: Stocks/Bonds (Private Company) Kimberley Brown, PharmD, ViiV Healthcare: Employee|ViiV Healthcare: Stocks/Bonds (Public Company) Maggie Czarnogorski, MD MPH, ViiV Healthcare: Employee|ViiV Healthcare: Stocks/Bonds (Private Company) Nanlesta Pilgrim, PhD, ViiV Healthcare: Employee|ViiV Healthcare: Stocks/Bonds (Private Company)

